# Interactive salinity and water stress severely reduced the growth, stress tolerance, and physiological responses of guava (*Psidium Guajava* L.)

**DOI:** 10.1038/s41598-022-22602-5

**Published:** 2022-11-08

**Authors:** Muhammad Mohsin Abrar, Muhammad Sohail, Muhammad Saqib, Javaid Akhtar, Ghulam Abbas, Hafiz Abdul Wahab, Muhammad Zahid Mumtaz, Khalid Mehmood, Muhammad Suleman Memon, Nan Sun, Minggang Xu

**Affiliations:** 1 Key Laboratory of Arable Land Quality Monitoring and Evaluation, Ministry of Agriculture and Rural Affairs, Institute of Agricultural Resources and Regional Planning, Chinese Academy of Agricultural Sciences, Beijing, 100081 China; 2 Institute of Soil and Environmental Sciences, University of Agriculture, Faisalabad, Pakistan; 3 Department of Environmental Science, COMSATS University Islamabad, Vehari Campus, Vehari, Pakistan; 4 Institute of Molecular Biology and Biotechnology, The University of Lahore, Lahore, Pakistan; 5 School of Environmental Science and Engineering, Nanjing University of Information Science and Technology, Nanjing, 210044 China; 6 Soil Fertility Research Institute, Agriculture Research Center, Tandojam, Sindh Pakistan; 7 Institute of Agricultural Environment and Resources, Shanxi University, Taiyuan, 030006 China

**Keywords:** Plant physiology, Plant stress responses, Plant sciences, Environmental impact

## Abstract

Salinity and water stress are serious environmental issues that reduced crop production worldwide. The current research was initiated (2012) in the wirehouse of the Institute of Soil and Environmental Sciences, University of Agriculture, Faisalabad, Pakistan to investigate the growth, stress tolerance, and physiological responses of guava to salinity and water shortage. Guava was grown for one year in pots containing soil with Eight treatments (control, 10 dS m^−1^, 20 dS m^−1^, 40 dS m^−1^, control + water stress (WS), 10 dS m^−1^ + WS, 20 dS m^−1^ + WS, 40 dS m^−1^ + WS) in a completely randomized design. The results indicated that plant growth, stress tolerance, and physiological parameters declined at higher salinity and water stress and could not survive at 40 dS m^−1^. The 20 dS m^−1^ + WS caused a > 70% decline in dry weights of shoot and root regarding control. Similarly, the highest decrease in stress tolerance was noticed in 20 dS m^−1^ + WS followed by the 20 dS m^−1^ treatment than control. Our findings validated that guava can be cultivated on soils having salinity ≤ 10 dS m^−1^ but it could not be cultivated on soils having salinity ≥ 20 dS m^−1^ with limited water supply.

## Introduction

Soil salinization is a major environmental problem that has affected more than 6% of the land area and about 20% of the irrigated soils globally^1^. Pakistan is also under the pertinent threat of soil salinization, where about 6.67 mha of the cultivated land area is affected by a certain level of soil salinization^2^. Soil salinity hampers the growth and productivity of many plants^3–5^. The osmotic effect is the first principal constraint imposed by salinity, which reduces the water uptake by plants^6^. The osmotic effect is followed by ion toxicity and nutrient imbalances in plants^7,8^. The salt tolerance mechanisms in plants include restricted uptake of unwanted ions (Na^+^ and Cl^−^), vacuolar compartmentation, ion exclusion^9^, translocation of toxic ions from shoots to roots, scavenging of reactive oxygen species (ROS)^10^, and maintenance of sufficient levels of K^+^ ions^11,12^ and better protection against oxidative stress^13^.

Water stress or drought is another serious issue of changing climate due to which up to 50% yield reduction in the field has been reported in many crops^14–16^. The different growth stages of plants are susceptible to water stress^17^. Like salinity stress, water stress also induces an osmotic effect^11^ and nutritional disorders in plants^12,16^. Additionally, many macromolecules such as carbohydrates, proteins, lipids, and chloroplast are damaged due to oxidative stress (generation of reactive oxygen species)^18–20^, in electron transport processes in chloroplasts and mitochondria, chlorophyll a, b and total contents^12^. Due to lipid peroxidation, the stability of cell membranes is decreased resulting in leakiness of osmolytes^12^. The plants adapt to water-scarce conditions by increasing stomatal resistance, maintaining high relative water contents in tissues, and absorbing more water through a widespread root system^21^.

*Psidium guajava* L., also called guava, is considered a very important fruit crop in many arid and semi-arid parts of the globe ^22^. Soil salinity and water shortage are very common issues in these areas. Mango tops the list of most important fruits in Pakistan, followed by citrus, apple, and guava. It makes guava the fourth most important fruit in Pakistan. According to Steppuhn et al.^23^, guava has been ranked as a moderately salt-tolerant plant. On the other hand, Ebert^24^ has categorized this fruit tree as very sensitive to salinity. Moreover, various growth stages of guava showed different responses to salinity stress. For example, the seed germination stage and seedling stage are relatively more prone to salt-induced damage than advanced growth stages^25,26^. Plant growth and physiological attributes are considerably declined when drought is imposed up to a 50% level of field capacity^27^. Since guava has considerable importance as a fruit as well as a medicinal plant^28^, therefore, the evaluation of morpho-physiological responses and investigation of tolerance of this tree to the interactive effects of salinity and water stress seems very important. Although numerous studies have already investigated the separate effects of salinity and water stress separately, however, there are very few studies existed that evaluated the interactive effects of salinity and water stress.

A plethora of literature is available regarding the responses of plants to certain levels of salinity as well as water stress separately. However, regarding natural field conditions, these two stresses occur together; therefore, the growth, stress tolerance, and physiological responses of plants should be studied under the combined stress conditions. Moreover, it has been noticed that the level of water in the soil greatly modulates the salt effects on plants^11,12^. Also, salt-affected arid regions often face water stress conditions. Therefore, the above-mentioned facts, the current study has been planned to unravel the growth, stress tolerance, and physiological responses of guava under the combined stress of salinity and limited water supply.

## Results

### Plant growth

**Figure 1:**
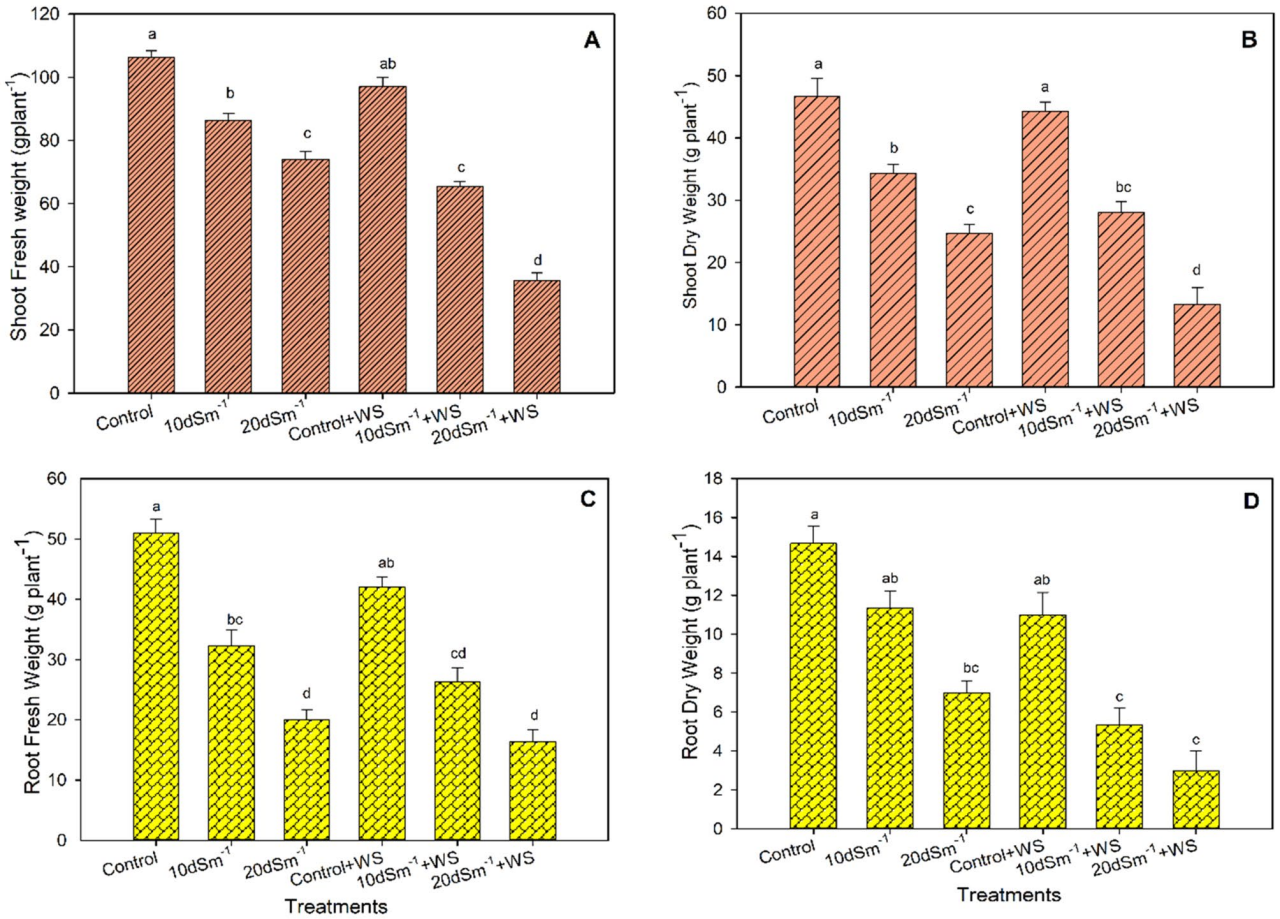
(**A**) Fresh weight and (**B**) dry weight of the shoot, and (**C**) fresh weight and (**D**) dry weight of the root of guava under salinity and water stress treatments. The different letters show significant differences according to the Tukey Honest Significant Difference (HSD) test after ANOVA (*p* ≤ 0.05); WS = water stress.

The plants growing under salinity level of 40 dS m^−1^ and interactive salinity and water stress treatment i.e., 40 dS m^−1^ + WS (water stress) did not survive in these high saline and drought conditions and died within three weeks. Therefore, their data are not available. Increasing levels of soil salinity significantly affected shoot and root growth parameters such as shoot fresh weight (SFW), root fresh weight (RFW), shoot dry weight (SDW), and root dry weight (RDW) of guava plants (Fig. 1A–D). However, water stress alone did not appreciably affect these attributes. Among the salinity treatments, the soil salinity level of 20 dS m^−1^ caused the highest reduction in shoot and root biomass compared with the control. Water stress further worsen the effect of high salinity levels on the shoot growth of guava. The combined treatment of high salinity level and the water stress (20 dS m^−1^ + WS) showed the most prominent decline in fresh and dry weights of the shoot (67% and 71%, respectively), in contrast to the control treatment. Both the fresh and dry root weights were decreased to a similar extent in the salt treatment of 20 dS m^−1^, alone and its combination with water stress (20 dS m^−1^ + WS). The reduction in root biomass was more than 40% in these treatments in comparison to the control.

**Figure 2:**
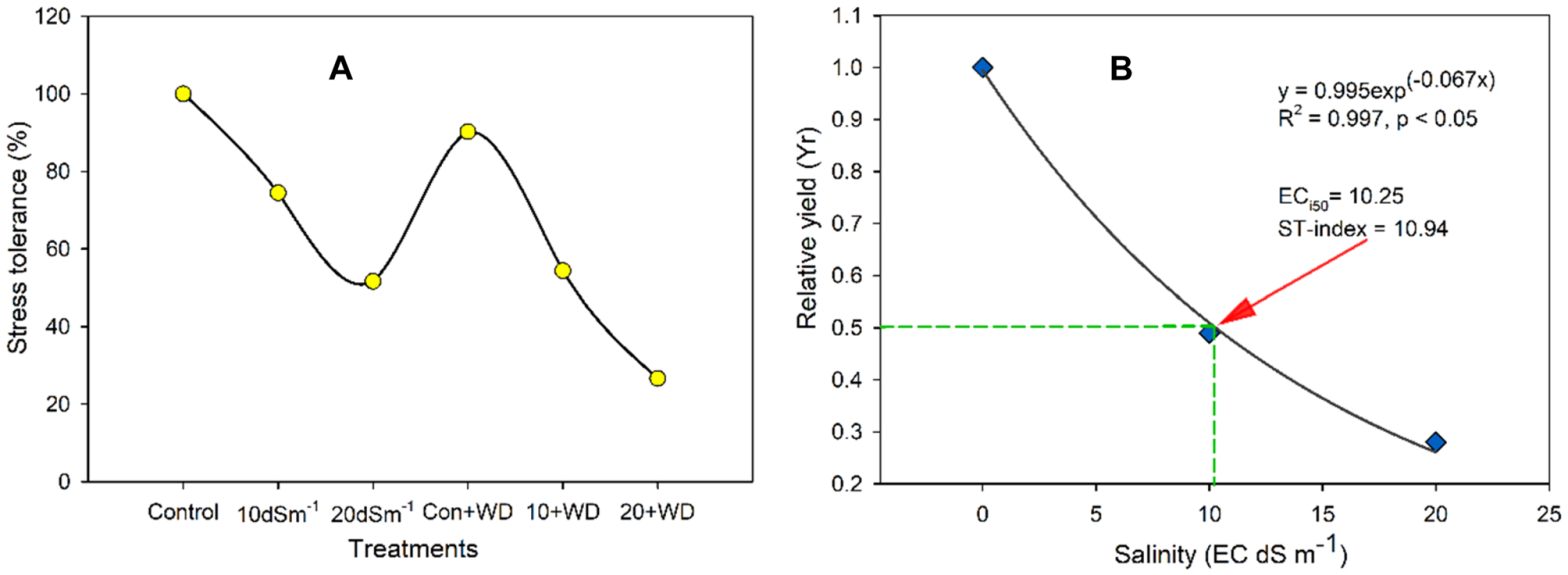
(**A**) stress tolerance (%) and (**B**) Salinity Tolerance Index (STI), Relative Yield (Yr) was based on the root and shoot dry masses (g) according to Steppuhn et al. (2005).

The combined treatment of salt and the water stress (20 dS m^−1^ + water stress) caused the highest decline in fresh and dry weights of the root (68% and 80%, respectively), in comparison to the control. Under 20 dS m^−1^ soil salinity level, fresh and dry weights of root were respectively decreased by 61% and 64% regarding control treatment. The second combined treatment of salinity and water stress conditions (10 dS m^−1^ + water stress) led to a 48% and 52% decline in fresh and dry weights of the root in contrast to the control treatment (*p* < 0.05). Similarly, the highest decrease in stress tolerance (73%) was noticed in 20 dS m^−1^ + WS followed by the 20 dS m^−1^ treatment where a 48% decrease in stress tolerance was found compared with control (Fig. 2B). An exponential decay model was employed between salinity (EC dS m^−1^) and relative total plant biomass (Y_r_) based on the best fitting of the data points (Fig. 2C).

### Chlorophyll contents and gas exchange attributes

**Figure 3:**
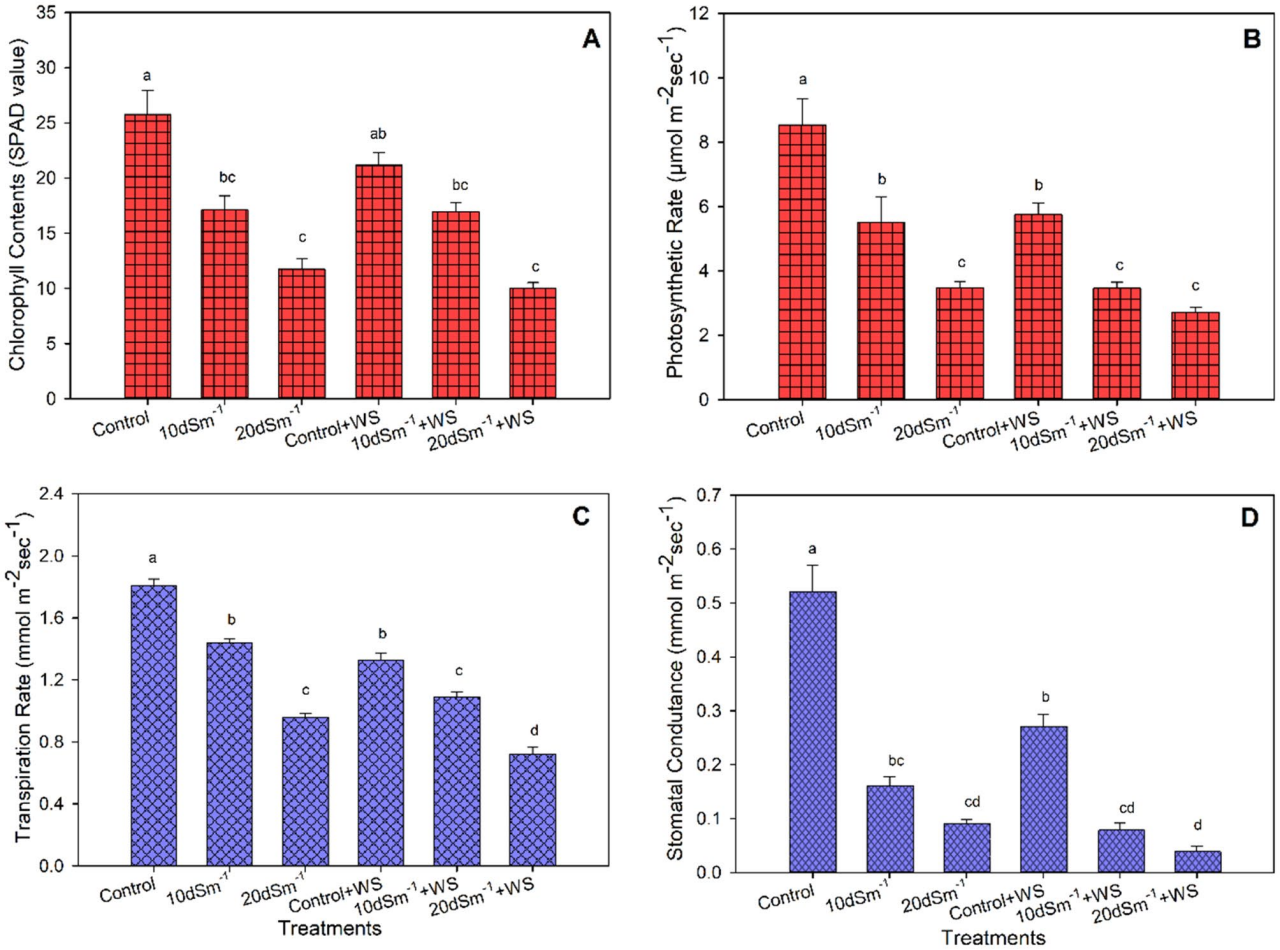
(**A**) Chlorophyll contents (**B**), photosynthetic rate (**C**), transpiration rate, and (**D**) stomatal conductance of *Psidium guajava* under various salinity and water stress treatments. The different letters show significant differences according to the Tukey HSD test after ANOVA (*p* ≤ 0.05); WS denotes Water stress.

Chlorophyll contents were depreciated only under salt stress while water stress has a non-significant effect on pigment contents in guava (Fig. 3A). Interestingly, the combined treatments of salinity and water stress caused a similar reduction in chlorophyll contents as did the salinity alone treatments.

Increasing levels of salinity had detrimental effects on photosynthetic rate, transpiration rate, and stomatal conductance (Fig. 3B–D). These attributes also significantly declined under water stress conditions as compared to control. The higher level of salinity alone (20 dS m^−1^) and two combined treatments of salinity and water stress (10 dS m^−1^ + WS; 20 dS m^−1^ + WS) had a similar effect on the photosynthetic rate and stomatal conductance of guava. However, the highest decrease in transpiration rate as compared to control (60%) was observed in the combined treatment of salt stress and water stress (20 dS m^−1^ + WS).

### Ionic contents

The concentration of Na^+^ was significantly higher in root, stem, and leaf when the guava plants were exposed to the combination of salt and water stress conditions (Table 1). The highest increase in Na^+^ concentration in the above-mentioned plant parts was observed under soil salinity treatment of 20 dS m^−1^ + water stress. The next highest level of Na^+^ was recorded in soil salinity treatment of 20 dS m^−1^ followed by the combined treatment of salt and water stress (10 dS m^−1^ + water stress).

**Table 1:** Regression between ionic contents (Na^+^ and K^+^) in shoot and root of guava plant (*Psidium Guajava* L.), and stress tolerance against salinity and water stress treatments.

Parameters	Regression	Coefficient of correlation (R)	Adjusted regression coefficient (Adj.R^2^)	Significance
Na^+^ in shoot vs. Stress tolerance	y = −22.6x + 103.6	0.849	0.704	****
Na^+^ in root vs. Stress tolerance	y = −179x + 118.7	0.980	0.959	****
K^+^ in shoot vs. Stress tolerance	y = 68.5x−29.06	0.974	0.945	****
K^+^ in root vs. Stress tolerance	y = 320x−31.98	0.946	0.889	****

Na^+^ and K^+^ denote sodium and potassium, respectively. Results are significant at *p* ≤ 0.0001 represented by ****. Regression is based on a first-order kinetics equation (y = bx + a) where “a” is the intercept, and “b” is the slope.

Soil salinity alone and in combination with water stress significantly reduced K^+^ concentration in the root, stem, and leaf (Table 1). The concentration of K^+^ in the afore-mentioned plant parts was noticed to be the highest in the control treatment. Contrarily, it was the lowest in the combined treatment of salinity and water stress conditions (20 dS m^−1^ + water stress), followed by a salinity treatment of 20 dS m^−1^ and a combined salinity and deficit supply of water (10 dS m^−1^ + water stress).

The highest K^+^/Na^+^ ratios (4.55, 2.85, and 14.33) in stem, leaf, and root were noted in the control, while the least K^+^/Na^+^ average values were found in 20 dS m^−1^ + water stress. Following the control, water stress showed K^+^/Na^+^ ratios of 4.22, 1.50, and 2.11 in the stem, leaf, and root, respectively. Nonetheless, a non-significant (*P* > 0.05) change in the K^+^/ Na^+^ ratio of the root was observed between 20 dS m^−1^, and 10 dS m^−1^ + water stress (Table 1).

### Relationships of Na^+^ content in shoot and root with stress tolerance

**Figure 4:**
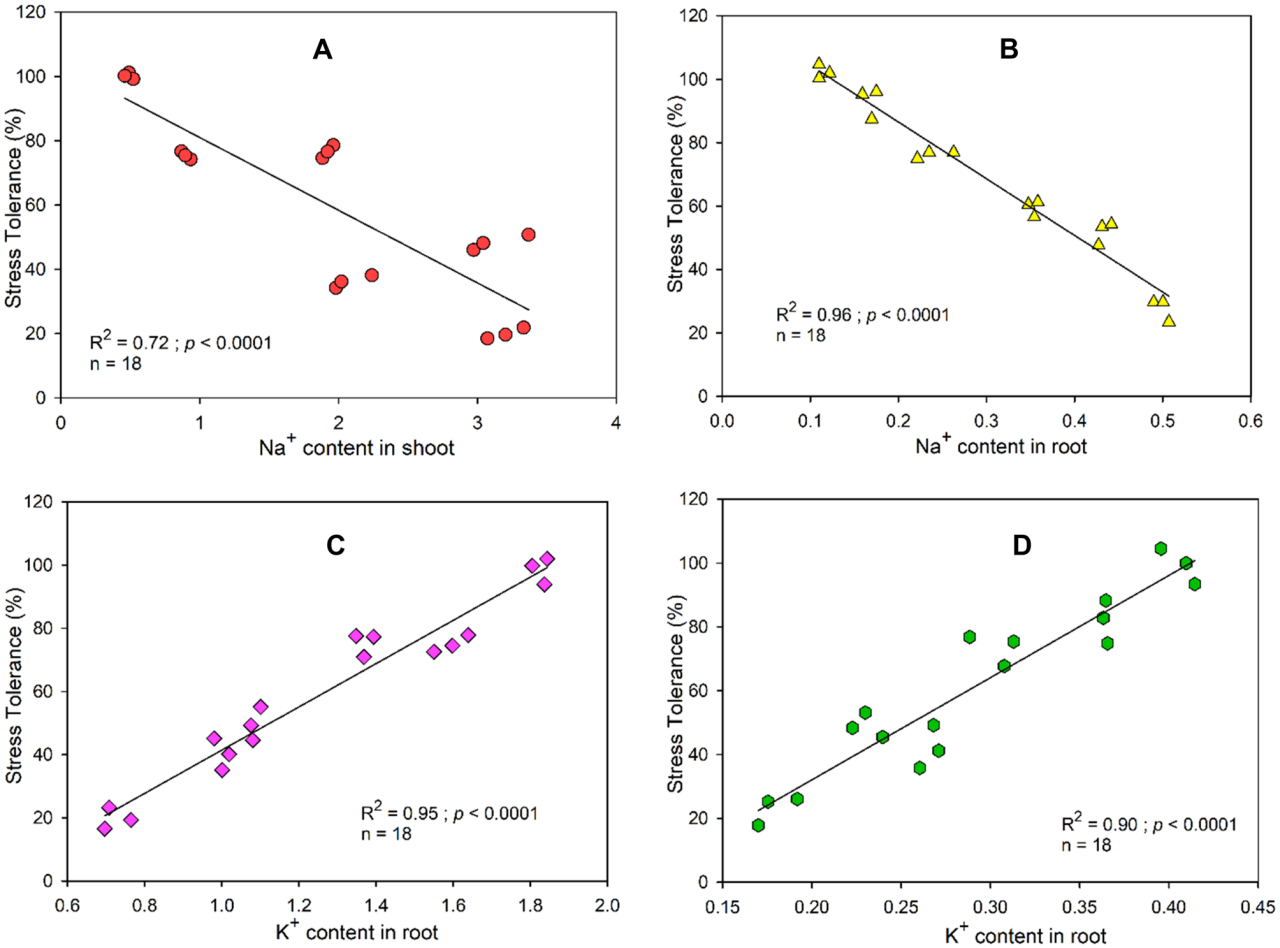
Linear regression of (**A**) sodium (Na +) content in the shoot to stress tolerance, (**B**) sodium (Na +) content in root to stress tolerance, (**C**) potassium (K +) content in the shoot to stress tolerance), and D) potassium (K +) content in the shoot to stress tolerance. All relationships are significant (*p* ≤ 0.05, 0.001, 0.001, 0.001, and 0.01, respectively).

Figure 4 shows that significant negative relationships are recorded between Na^+^ content in shoot and root, and stress tolerance. For instance, Na^+^ content in the shoot explains a 73% variance in stress tolerance. While increasing Na^+^ content in the root explains 96% variability in salinity stress (Fig. 4B). In contrast, K^+^ content in shoot and root, respectively showed positive correlations (R = 0.974; R = 0.918; Table 1) with stress tolerance (Fig. 4C,D).

### Relationships of K^+^/Na^+^ ratio in leaf, stem, and root with biomass and physiological attributes

**Figure 5:**
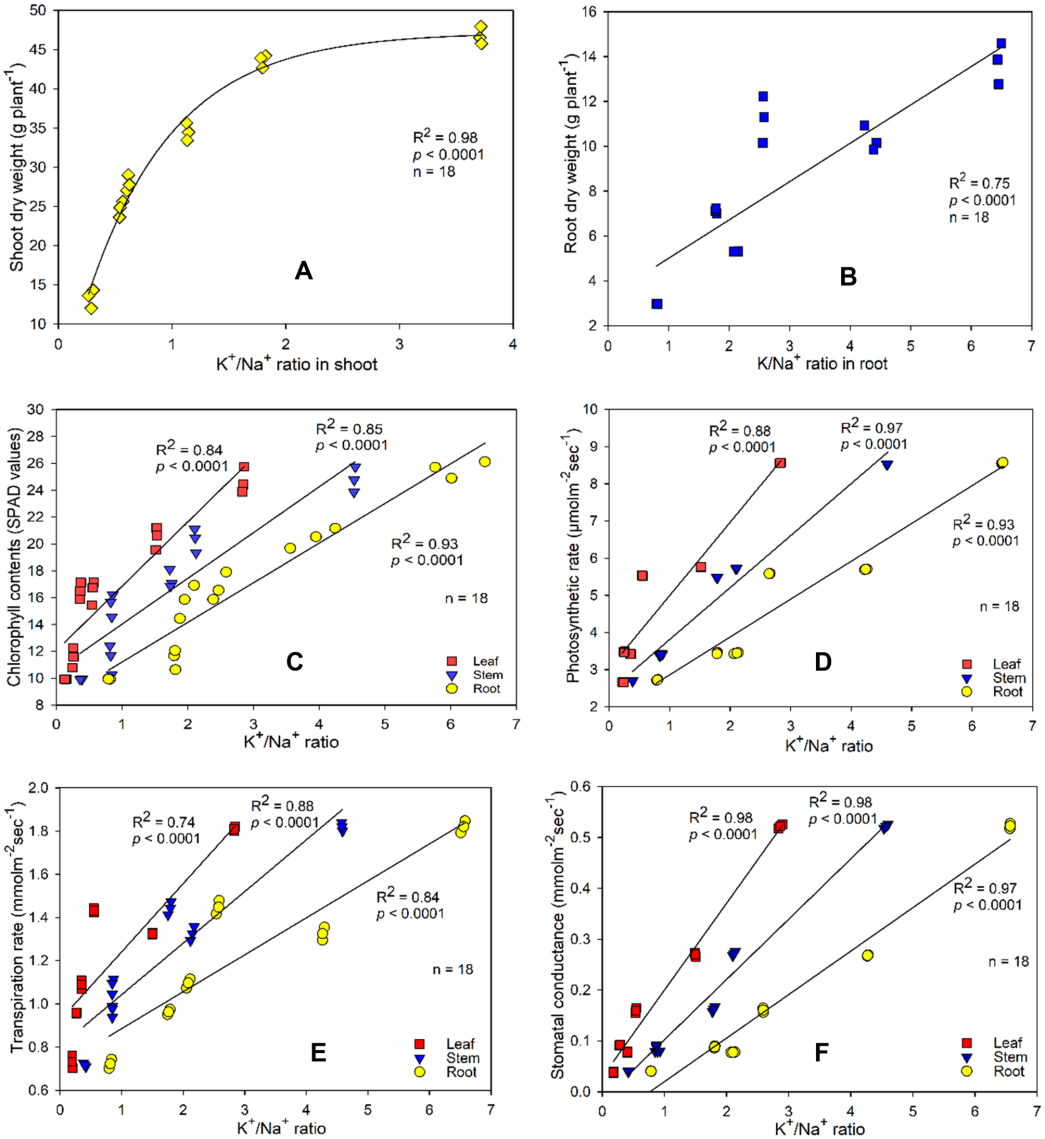
Relationship between K^+^/Na^+^ ratio and (**A**) Shoot dry weight and (**B**) Root dry weight, and physiological attributes (**C**) Chlorophyll content (**D**) Photosynthetic rate (**E**) Transpiration rate and (**F**) Stomatal conductance. The coefficient of regression is denoted by “R^2^” and “*p”* represents the level of significance.

An exponential model was observed between the K^+^/Na^+^ ratio in shoot and SDW (Fig. 5A) while a linear model was recorded between the K^+^/Na^+^ ratio in root and RDW (Fig. 5B). Similarly, highly significant regressions were observed between the K^+^/Na^+^ ratio (leaf, stem, and root) and physiological attributes (Fig. 5C–F). For instance, a clear relationship between K^+^/Na^+^ ratio and chlorophyll content, and K^+^/Na^+^ ratio and photosynthetic rate with the slope and regression values (b = 5.11; R^2^ = 0.834) in leaf, stem, and root, respectively. Similarly, in Fig. 5E,F the highest slope values of 0.316 and 0.170 were observed in regressions between the K^+^/Na^+^ ratio and transpiration rate (leaf), and K^+^/Na^+^ ratio and stomatal conductance (leaf), respectively (Table 2).

**Table 2:** Regression between ionic contents (Na^+^ and K^+^) in shoot and root of guava plant (*Psidium Guajava* L.), and stress tolerance against salinity and water stress treatments.

Parameters	Regression	Coefficient of correlation (R)	Adjusted regression coefficient (Adj.R^2^)	Significance
K^+^/Na^+^ (shoot) vs. shoot dry weight	y = 47.20^−exp(−1.31x)^	0.990	0.980	****
K^+^/Na^+^ (root) vs. root dry weight	y = 1.911x + 3.000	0.865	0.732	****
K^+^/Na^+^ vs. chlorophyll content (leaf)	y = 5.105x + 12.26	0.836	0.825	****
K^+^/Na^+^ vs. chlorophyll content (stem)	y = 3.533x + 10.93	0.923	0.842	****
K^+^/Na^+^ vs. chlorophyll content (root)	y = 2.735x + 8.931	0.964	0.925	****
K^+^/Na^+^ vs. photosynthetic rate (leaf)	y = 1.940x + 3.063	0.938	0.872	****
K^+^/Na^+^ vs. photosynthetic rate (stem)	y = 1.401x + 2.454	0.985	0.968	****
K^+^/Na^+^ vs. photosynthetic rate (root)	y = 1.014x + 1.874	0.964	0.925	****
K^+^/Na^+^ vs. transpiration rate (leaf)	y = 0.316x + 0.924	0.861	0.724	****
K^+^/Na^+^ vs. transpiration rate (stem)	y = 0.238x + 0.808	0.936	0.868	****
K^+^/Na^+^ vs. transpiration rate (root)	y = 0.173x + 0.708	0.914	0.826	****
K^+^/Na^+^ vs. stomatal conductance (leaf)	y = 0.170x + 0.032	0.991	0.981	****
K^+^/Na^+^ vs. stomatal conductance (stem)	y = 0.118x–0.012	0.992	0.980	****
K^+^/Na^+^ vs. stomatal conductance (root)	y = 0.086x–0.065	0.986	0.970	****

Na^+^ and K^+^ denote sodium and potassium, respectively. Results are significant at *p* ≤ 0.0001 represented by ****y. All regressions are based on first order kinetics equation (y = bx + a) where “a” is the intercept, and “b” is the slope value for the regression except regression between K^+^/Na^+^ (shoot) and shoot dry weight is based on exponential model (y = a^−exp(bx)^).

### Principal component analysis

**Figure 6:**
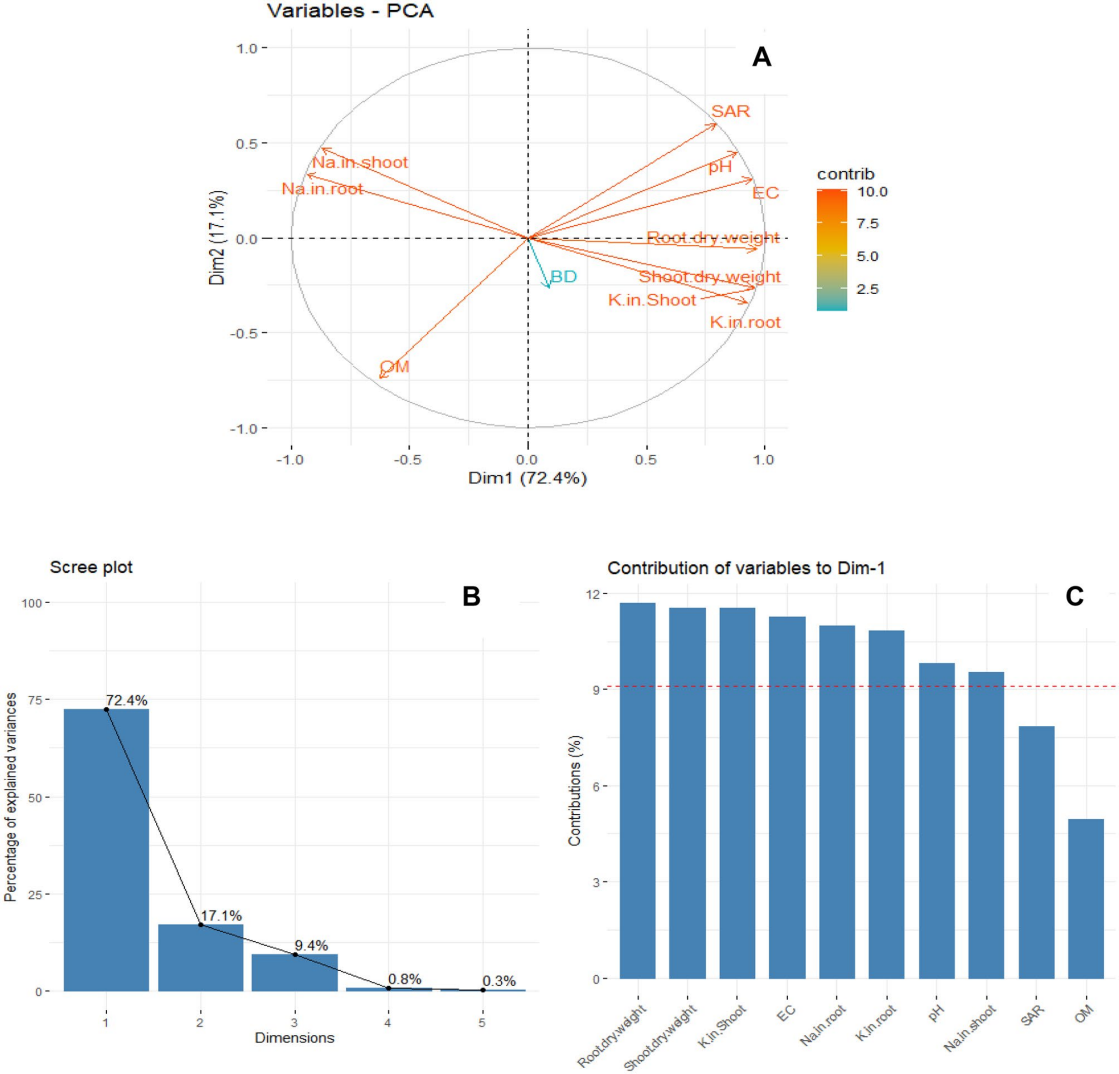
Principle component analysis (PCA) of soil physico-chemical properties, plant growth attributes, and ionic contents. (**B**) Percent variance explained by each dimension of PCA. The individual variability (%) explained by each dimension (Dim1 to Dim 5) was presented on each bar. C) Percent contribution of each variable to the first two dimensions of PCA. The dashed line depicts ~ 90% contribution to the significant component.

The first two Principal Components axes (PC1 and PC2) explained 90% of the total variability. The shoot dry weight, root dry weight, K^+^ in the root, and K^+^ in the shoot correlated positively with pH, and BD, and negatively with Na^+^ in the root and Na^+^ in the shoot (Fig. 6). The PC1 and PC2 axes explained 72.4% and 17.1% of the total variability. We found that ionic contents (Na^+^ in the shoot and root had highly significant negative correlations with K^+^ in the shoot and K^+^ in the root, respectively. Similarly, SOM and BD also had a negative relationship. While EC of the soil had positive associations with the shoot and dry weights (Fig. 5; Tables S1 and S2 Supplementary materials). Na^+^ contents in shoot and root were negatively correlated with the shoot and root dry weight, and K^+^ concentration in shoot and root, respectively. Similarly, OM was negatively associated with SAR, pH, BD, and EC (Fig. 6A). While shoot and root dry weight and K^+^ concentration of shoot and root were positively correlated with each other. Moreover, no significant relationships were observed among BD, pH, EC, and SAR (Fig. 6A).

## Discussion

### Plant biomass

The current study was designed to evaluate the influence of water stress on the salt tolerance ability of guava. The values of EC_i50_ and ST-indexes (10.25 dS m^−1^; 10.94, respectively), indicated that *Psidium guajava* can be considered as moderately tolerant to salinity (Fig. 2C) Steppuhn et al. (2005a) as compared to other fruit crops, such as the *Jatropha curcas* (STI-index = 11.4; based on shoot and root growth)^3^, and some fruit plants such as almond (EC_i50_ = 3.83 dS m^−1^, ST-index = 4.94; based on shoot growth), apricot (EC_i50_ = 3.39 dS m^−1^, ST-index = 4.63; based on shoot growth), and date palm (EC_i50_ = 17.42 dS m^−1^, ST-index = 18.38; based on fruit yield) as noted by Steppuhn et al*.* (2005a). Our findings revealed that water stress alone was not as much detrimental to the plant growth of guava (Fig. 1). However, the combined application of water stress with the higher salinity level (20 dS m^−1^ + WS) was more damaging than salinity alone treatment (20 dS m^−1^), for fresh and dry weight of shoot. The effect of the combination of both these treatments was not additive in the case of root biomass of *Psidium guajava*. Our results demonstrated that *Psidium guajava* could not survive at the higher salt level (40 dS m^−1^), either alone or with a limited water supply. Likewise, at 20 dS m^−1^, a drastic drop in plant biomass was noticed. However, the plant produced reasonable biomass at 10 dS m^−1^ + water stress, which indicates that this important fruit plant can be grown on moderately salt-affected soil under water-scarce conditions. Many previous studies had shown that the plant biomass decreases as the plants face salinity^3,11,12^, and water stress conditions^16,29^, or their combination^3^.

Abbas et al. (2016) reported that the plant biomass of two acacia species was significantly decreased when plants were grown under salinity and water stress. In the present study, we employed stress tolerance (%) as indices of plant biomass, which were considerably depreciated in the combined salinity and water stress conditions (Fig. 2A). Salinity-induced growth reduction can be attributed to ion toxicity, osmotic effect, and nutrient deficiency^3,8,11^. Similarly, limited water supply to plants may cause osmotic problems^11^, and deficiency of nutrients in most plants^16^. The above-mentioned adverse effects operate at the cellular and whole plant level, thereby decreasing plant growth and biomass production. Osmotic effects and perhaps nutrient deficiency effects caused by water stress augmented the harmful effects of salt stress (Fig. 3 and Table 3). As a result of additive effects, guava plants grown at the same salinity level but having a shortage of water produced less biomass (Fig. 1).

**Table 3:** Concentrations of Na^+^, K^+^, and K^+^/Na^+^ ratio in *Psidium guajava* exposed to the salt (NaCl) and drought stress (low water supply). Data refer to mean values (n = 3). The same letters along with columns are not significantly different according to the Tukey HSD post hoc test after ANOVA (*p* ≤ 0.05).

Treatments	Na^+^ (mmol g^−1^dw)			K^+^ (mmol g^−1^dw)			K^+^/Na^+^ ratio		
	Leaf	Root	Stem	Leaf	Root	Stem	Leaf	Root	Stem
Control	0.46^d^	0.06^d^	0.11^d^	1.31^a^	0.39^a^	0.50^a^	2.85^a^	6.50^a^	4.55^a^
10 dSm^−1^	1.79^b^	0.12^bc^	0.22^bc^	0.96^b^	0.31^b^	0.39^b^	0.54^c^	2.58^c^	1.77^b^
20 dSm^−1^	2.91^a^	0.13^b^	0.36^b^	0.82^bc^	0.23^c^	0.30^c^	0.28^de^	1.77^cd^	0.83^c^
Control + WS	0.80^c^	0.09^c^	0.19^c^	1.20^a^	0.38^b^	0.40^a^	1.50^b^	4.22^b^	2.11^b^
10 dSm^−1^ + WS	1.89^b^	0.13^b^	0.35^b^	0.70^c^	0.27^c^	0.30^c^	0.37^cd^	2.08^cd^	0.86^c^
20 dSm^−1^ + WS	3.02^a^	0.16^a^	0.49^a^	0.50^d^	0.13^d^	0.19^d^	0.17^e^	0.81^d^	0.39^c^

D = Water stress; dw = dry weight; dSm^−1^ = desiSiemens per meter.

### Chlorophyll contents and gas exchange attributes

Leaf chlorophyll contents were decreased under salinity stress in guava. In line with our results, pigment contents of Acacia^30^, and soybean^31^ were decreased under salinity stress conditions. Salt stress conditions trigger the formation of the reactive oxygen species, which are known to have deleterious effects on chloroplast^20,32,33^.

It has been reported that under limited water supplies, stomata are closed to conserve moisture in the leaves; resultantly, stomatal conductance is decreased^16,34^. Reduced stomatal conductance under salinity or drought leads to a corresponding decrease in photosynthesis^35^, and transpiration rates^36^. Adequate stomatal conductance is crucial for maintaining water balance in the cell. In severe conditions of water deficiency, the activities of some essential enzymes including ribulose-1,5-bisphosphate carboxylase/oxygenase, and rubisco activase are inhibited, leading to a reduction in CO_2_ fixation^37^. According to Munns^38^, salinity stress may cause a decrease in the water uptake potential of plants, leading to a decrease in transpiration rate. We found that when guava was grown at the salt treatment of 10 dS m^−1^ with or without water stress conditions, these attributes were mostly less affected, again indicating the suitability of this tree plant to grow on saline soils (from slightly to moderately) successfully.

### Relationships of Na^+^ content and K^+^/Na^+^ ratio with stress tolerance, and morpho-physiological attributes

The Na^+^ concentration was increased under salinity stress, whereas the K^+^ concentration was decreased in the root and shoot of guava. Further, an increase in the concentration of Na^+^, on the contrary, a decrease in the concentration of K^+^ was recorded upon the combined application of salinity and water stress conditions. Through PCA, we also noticed negative associations between Na^+^ content in shoot and root and K^+^ content in shoot and root (Fig. 6A). Moreover, an increase in salinity in the soil matrix or within plant tends to decrease the K^+^ content in plant organs such as shoot or root (Fig. 6A). This ionic imbalance is well documented in plants when grown under salinity stress^8,12^ and a blend of salt and water stress conditions^3,11,12^. In this research work, results showed that guava leaves had the highest Na^+^ concentration, followed by stem and roots. Different plant species/genotypes have different Na^+^ uptake and sequestration potential. Some plant species exclude Na^+^ at the root level, while others accumulate and sequester excessive Na^+^ in their leaf vacuoles^6,12,38^. The Na^+^ uptake behavior of guava indicated that the prominent salt tolerance mechanism in this plant is the vacuolar sequestration of Na^+^.

In contrast to Na^+^ ions, the uptake of K^+^ ions was reduced when plants were grown under combined treatments of salinity and water stress^3,8,11,39^. Potassium has a vital role both in the salt and drought resistance of plants. Under the combined application of salt and water stress, the uptake of Na^+^ ions in the shoot was greater as compared to a decrease in plant essential K^+^ ions, which might have contributed to the lower salt tolerance potential of guava at a higher salinity level^12^. This greater accumulation of Na might be due to higher concentrations of Na ions in the saline soil.

Plants prefer to keep a higher K^+^ concentration compared with Na^+^ in stem and root. Various studies reported that plants try to acquire enough K^+^ concentrations that could actively ameliorate the adverse impacts of Na^+^ in tissues^40–42^. In contrast, the increased leaf Na^+^ content related to the decreased leaf K^+^ content in salinity treatments depreciates the K^+^/Na^+^ ratios. Our findings revealed that the K^+^/Na^+^ ratio declined with an increment in salt content (Table 1). Furthermore, potassium is an essential element for activating more than 50 enzymes^43^ including those enzymes responsible for biologically synthesizing the chlorophyll. Likewise Na^+^, the K^+^ ion has an identical ionic radius thus in higher salinity, Na^+^ ions via K^+^ channels enter the cell^44^. The higher Na^+^ concentration in cytoplasm results in a lowering K^+^/Na^+^ ratio, which consequently limits plant metabolism. Therefore, the plants’ ability to minimize K^+^ loss and maintain a higher cytoplasmic K^+^/Na^+^ ratio is an expression of their salt tolerance potential^45^. In this study, we observed a significantly elevated K^+^/Na^+^ ratio in the control, in comparison to the other treatments (Table 1). The least K^+^/Na^+^ ratio under 20 dSm^−1^ + WS also limited the physiological attributes such as photosynthetic and transpiration rates, chlorophyll contents, and stomatal conductance by 68%, 60%, and 61%, 92%, respectively, compared with control (Fig. 3 and Table 1). Linear regressions between the K^+^/Na^+^ ratio and physiological parameters also revealed that all the physiological parameters increased with increasing K^+^/Na^+^ ratio (Fig. 5). For instance, in agreement with our findings, Al-Karaki^46^ reported that retention of higher amounts of K^+^/Na^+^ ratio in leaf enhances salt tolerance in tomato. Moreover, Giri et al.^47^ concluded that enhanced tolerance to salinity may partially be attributed to the increased K^+^/Na^+^ ratios in shoot and root.

## Conclusions

It can be concluded that higher salinity stress (20 dS m^−1^) drastically reduced plant growth, chlorophyll contents, and leaf gas exchange attributes of guava plants. Plants could not survive at a salinity level of 40 dS m^−1^ due to severe salt stress. The effect of water stress was non-additive except for shoot biomass. Increased accumulation of Na^+^ and limited uptake of K^+^ concentration greatly contributed to lowering the salt tolerance of guava plants. The results demonstrated that guava is a moderately salt-tolerant plant and can be cultivated on saline soils having salinity levels up to 10 dS m^−1^ under sufficient water supply. However, the cultivation of this fruit tree is not recommended on soils having higher salinity level (20 dS m^−1^) accompanied by limited water supply Nevertheless, these findings need further validation on saline soils prone to drought stress under actual field conditions.

## Materials and methods

### Site description, treatments, and plant establishment

The impact of salt and water stress on the growth and physiological parameters of guava was investigated. The pot experiment was conducted in a rain-protected wirehouse of the Institute of Soil and Environmental Sciences (ISES), University of Agriculture Faisalabad (UAF), Pakistan. The protection from the rain was achieved by a polycarbonated sheet used as the roof of the wirehouse. Non-saline (ECe = 2.30 dS m^−1^) sandy clay loam soil was taken from the field of ISES. The collected soil was dried in the open air and subsequently sieved through a 2 mm sieve. Then, 12 kg of this soil was filled per plastic pot. Different salinity levels, i.e., 10, 20, and 40 dS m^−1^ were developed in the soil of each pot by properly mixing NaCl salt. The NaCl was added based on the difference between the initial EC and the required EC of the soil. The required TTS were multiplied by the equivalent weight of NaCl and the given amount of NaCl was applied to the soil. The soil was kept for two months for equilibrium before transplanting the seedlings. These salinity levels were calculated based on saturated soil extract ECe. Pots without salt addition served as control. Eight treatments of salinity and water stress (control, 10 dS m^−1^, 20 dS m^−1^, 40 dS m^−1^, control + water stress, 10 dS m^−1^ + water stress, 20 dS m^−1^ + water stress, 40 dS m^−1^ + water stress) were used in the study. In water stress treatments, plants remained under water stress for 15 days (no irrigation), and 15 days of normal watering (irrigation twice a week). The plants that did not receive water stress treatments were regularly irrigated twice a week to maintain the moisture level (70%, water holding capacity). One liter of water was applied for reirrigation. Initially, they were acclimatized for one month, and water stress treatments were used afterward. These treatments were continued until the harvesting of the plants. Each treatment was replicated four times, and the pots were randomly arranged in the wirehouse. Uniformly growing guava plants (cultivar ‘Safeda’) at the age of three months (since seed germination; with about 20 cm plant height) were shifted to pots (one plant per pot). The plants were grown and acclimatized for three months before transplanting. On the off chance, when a plant died during the first month, it was replaced with a new plant having the same size and age, just to let it establish in stress conditions. The soil used for the pot experiment was analyzed for different physico-chemical soil properties (Table 3) according to the method proposed by Ryan et al.^48^.

### Plant growth

The plants were harvested after one year, and subsequently, the data related to growth parameters such as fresh weight (shoot and root), and dry weight (shoot and root) were recorded. The shoot and roots were separately harvested and weighed for fresh weight. Afterward, the samples were oven-dried, and their dry weights were recorded**.**

### Chlorophyll and gas exchange attributes

Leaf chlorophyll content (SPAD value) of the completely grown second leaf from the top of each plant was measured with the help of a SPAD-502 chlorophyll meter. Three leaves per plant were used for taking the readings with one reading per leaf. In contrast, the leaf gas exchange parameters including stomatal conductance, transpiration rate, and photosynthetic rate of the same leaf, were measured from sampled leaves with the help of an LCA-4 ADC transportable infrared gas analyzer (ADC BioScientific Ltd., Global House, Geddings Road, Hoddesdon, Herts EN11 0NT, UK). The above-mentioned parameters were recorded one day before harvesting on a sunny day for about two hours around noon. The data were recorded under these instrument conditions: temperature in the leaf chamber was 34.5 to 39.6 °C, the ambient concentration of CO_2_ (C_ref_) was about 373 μmol mol^−1^, ambient pressure was about 991 mBar, the sub-stomatal concentration of CO_2_ (Ci) ranged from 115 to 312 μmol mol^−1^, leaf surface PAR (Q leaf) was 1200 μmol m^−2^ s^−1^, and the average water vapor pressure of the chamber was 34.6 mBar.

### Ionic contents

The root, stem, and leaf samples were individually oven-dried at 70 °C for 48 h; then were finely ground and subjected to wet digestion in H_2_SO_4_ and H_2_O_2_ as described by Wolf^49^. After digestion, the digestate was filtered, and with distilled water, the final volume of 50 mL was developed for the measurement of ions. The flame photometer (Sherwood-410, 1 The Paddocks, Cherry Hinton Road, Cambridge, CB1 8DH UK) was used for measuring Na^+^ and K^+^ concentrations in leaf, root, and stem samples.

## Calculations

### Stress tolerance

Stress tolerance is the simple difference between the trait measured in control and stress conditions^50^. Selection based on Stress tolerance favors genotypes with higher mass under stress conditions (compared to control).

### Salinity tolerance index

Total root and shoot dry masses were used (in terms of yield) to model the response functions of guava to applied salt and drought treatments^23^. The total dry mass (Y) was first calculated as relative dry mass (Y_r_) to compare the obtained data by using a scaling divisor (Y_m_) based on the highest amount of total biomass under non-treated conditions^51^. Each Y_r_ value has been calculated by the Eq. (1):

1 $${Y}_{r}=\frac{Y}{{Y}_{m}}$$

To check the response of dry mass to salinity, next to the data transformation according to Eq. (1), an exponential model was used as a function of the best-fit line and the highest R^2^:

2 $${Y}_{r}=a\times {e}^{b\times {EC}_{i}}$$

Here EC_i_ represents the irrigation water electrical conductivity; “a” reflects the curve shape and is a constant, and “b” defines the model intensity and is negative always. The salt tolerance index (ST-index) shows the inherited tolerance of crops to salinity in the root zone.

If Yr = 0.5, the irrigation water electrical conductivity for dry mass reduction of 50% of the maximum dry mass (EC_i50_) is gained from Eq. (3). The ST-index can be calculated by the following equation adapted from Steppuhn et al. ^52^:

3 $$ST-index={EC}_{i50}\times (1+b)$$

### Statistical analysis

Each treatment was repeated thrice. The data were analyzed following standard procedure in a completely randomized design (CRD) with one-way ANOVA at a significance level of *p* < 0.05. The least significant differences between mean values were determined by Tukey Honest Significant Difference (HSD) posthoc test. All of these statistical analyses were performed by using IBM-SPSS 23.0 software (SPSS Inc. Chicago, IL., USA). Principal component analysis (PCA) was employed to evaluate the relationships among soil physico-chemical properties, plant growth attributes such as shoot and root dry weights, ionic contents e.g., Na^+^ content in shoot and root, and K^+^ content in shoot and root using the princomp function of the factoextra and factoMineR packages of R software (Version 4.1.0) (R Core Team, Vienna, Austria). The PCA biplot, contribution plot and eigenvalues corresponding to the variation explained by each principal component were performed using the fviz function of factoextra.

## Supplementary Information

Supplementary Information.

## Data Availability

The datasets used and/or analyzed during the current study are available from the corresponding author upon reasonable request.
