# Supplementary material for: Interactive salinity and water stress severely reduced the growth, stress tolerance, and physiological responses of guava (Psidium Guajava L.)
Source: Sci Rep. 2022 Nov 8;12:18952. doi: 10.1038/s41598-022-22602-5 (PMC9643515; doi:10.1038/s41598-022-22602-5)
Supplement: Supplementary file 1 — Supplementary Information. [file 41598_2022_22602_MOESM1_ESM.docx]

**Supplementary Materials**

**Table S1** Basic physico-chemical properties of soil (sampled from the nearby field plot) used in the experiment

| **Properties** | **Value** | **Unit** |
| --- | --- | --- |
| Textural class | Sandy clay loam | None |
| Bulk density (BD) | 1.52 | (gcm^-3^) |
| Silt | 33 | % |
| Clay | 18 | % |
| Sand | 49 | % |
| pHs | 7.35 | None |
| SAR | 7.3 | (mmolL^-1^)^1/2^ |
| Saturation percentage | 28 | % |
| Soil organic matter (SOM) | 0.68 | % |
| Electrical conductivity of soil saturated extract (ECe) | 2.30 | dS m^-1^ |
| Available Phosphorous (AP) | 3.43 | mg kg^-1^ |
| Soluble Na^+^ | 18.13 | (mmolc L^-1^) |
| Soluble K^+^ | 1.43 | (mmolc L^-1^) |
| Soluble CO_3_^2-^ | 0.85 | mmol_c_ L^-1^ |
| Soluble HCO_3_^-^ | Nil | mmol_c_ L^-1^ |
| Cation exchange capacity (CEC) | 16.89 | cmol_c_ kg^-1^ |
| Exchangeable Na^+^ | 1.02 | % |
| Exchangeable K^+^ | 0.0145 | meqL^-1^ |

**Table S2** Principle component analysis (PCA) of soil physico-chemical properties, plant growth attributes, and ionic contents

| **Principal Component Number** | **Eigenvalue** | **Percentage of Variance (%)** | **Cumulative (%)** |
| --- | --- | --- | --- |
| Dim.1 | 8.80 | 80.0256700 | 80.02 |
| Dim.2 | 1.98 | 18.03 | 98.06 |
| Dim.3 | 0.11 | 1.03 | 99.08 |
| Dim.4 | 0.05 | 0.510 | 99.59 |
| Dim.5 | 0.04 | 0.40 | 100.00 |

Type: Correlation

**Table S3** Effect of salinity and water stress treatments on variations in absolute and relative yield parameters

| **Treatments** | **Absolute yield (Y)** | **Relative yield (Yr)** | **Max. yield (Ym)** |
| --- | --- | --- | --- |
| Control | 61.33 | 0.98 | 62.67 |
|  | 62.67 | 1.00 |  |
|  | 60.36 | 0.97 |  |
| 10 dSm^-1^ | 45.86 | 0.73 |  |
|  | 46.49 | 0.74 |  |
|  | 44.66 | 0.71 |  |
| 20 dSm^-1^ | 31.67 | 0.51 |  |
|  | 30.34 | 0.48 |  |
|  | 32.38 | 0.52 |  |
| Control+WD | 55.23 | 0.88 |  |
|  | 54.66 | 0.87 |  |
|  | 53.75 | 0.86 |  |
| 10 dSm^-1^+WD | 33.33 | 0.53 |  |
|  | 32.18 | 0.51 |  |
|  | 31.84 | 0.51 |  |
| 20 dSm^-1^ +WD | 16.73 | 0.27 |  |
|  | 15.66 | 0.25 |  |
|  | 14.95 | 0.24 |  |

dS m^-1^ = Decisiemens per meter; WD = water deficit

Absolute yield is expressed as dry matter produced in grams (g)
